# The difference between young and older ducks: Amino acid, free fatty acid, nucleotide compositions and breast muscle proteome

**DOI:** 10.1016/j.fochx.2024.102117

**Published:** 2024-12-22

**Authors:** Tiantian Gu, Mingcai Duan, Li Chen, Yong Tian, Wenwu Xu, Tao Zeng, Lizhi Lu

**Affiliations:** Key Laboratory of Livestock and Poultry Resources (Poultry) Evaluation and Utilization, Institute of Animal Husbandry and Veterinary Science, Zhejiang Academy of Agricultural Science, Hangzhou 310021, China

**Keywords:** Duck, Amino acid, Free fatty acid, Nucleotide, Proteome

## Abstract

Duck meat has a unique taste and nutritional value, but age probably affects meat quality. In this study, ducks of different ages (60-day-old, D60; 900-day-old, D900) were chosen, and the odor, taste, amino acid, nucleotide, and free fatty acid components of breast meat were evaluated to investigate the differences. The results showed that the amino acid contents of breast muscle in D900 ducks, especially in Asp (umami) and Thr (sweet), were richer than those in D60 ducks. In addition, the levels of guanosine-5′-monophosphate (GMP), inosine-5′-monophosphate (IMP), monounsaturated fatty acid (MUFA; C18:1 t), and polyunsaturated fatty acid (PUFA; C18:2) in D900 ducks were higher than those in D60 ducks. Proteomic approach was further performed to analyze the difference of breast muscle between young and older ducks in protein level. We found that 496 differentially expressed proteins (DEPs) were screened. GO and KEGG analysis mainly enriched in glycine, serine, and threonine metabolism, tyrosine metabolism, and pyruvate metabolism. Moreover, correlation analysis revealed that BPGM, ADH5, ME2, ME3, GLO1, and PDHB, were specifically correlated with amino acids, nucleotides, and free fatty acids in meat from D60 ducks, whereas only two proteins, GRHPR and COMT, showed specific correlations with amino acids, nucleotides, and free fatty acids in meat from D900 ducks. This study proposes several candidate protein biomarkers for older duck meat that should be evaluated in the future.

## Highlights

• 900-day-old ducks have better umami and sweet.
• 900-day-old ducks have higher monounsaturated and polyunsaturated fatty acids.
• Proteome revealed that DEPs were mainly enriched in the meat flavor metabolism.

## 1 Introduction

Duck meat is uniquely tasty and rich in digestible proteins, polyunsaturated fatty acids, vitamins, and inorganic elements (Bai et al., 2020), becoming widely popular among consumers and human consumption of high-quality duck meat. Evidences have been demonstrated that age is closely associated with the deposition of flavor substances and nutritional composition (Liu et al., 2013). Liu et al. reports that the meat of 230-day-old chickens has advantages in creatine, glucose, taurine, glutamine, and other compounds over meat from chickens of younger ages (Xiao et al., 2019). Further, in geese, total essential amino acids (TEAA) and polyunsaturated fatty acids (PUFA) in the meat are rich in increasing animal age (Weng et al., 2022). Regarding the influence of age on the flavor composition of duck meat, aromatic hydrocarbons, hexanal, and 2,3-octanedione are more abundant in older ducks (Liu et al., 2013).

Currently, proteomic strategies are being widely adopted to evaluate food quality (López-Pedrouso et al., 2019; López-Pedrouso et al., 2023). This can provide an in-depth characterization of molecular pathways and identify protein biomarkers to predict the quality of animal meat. For example, Doherty et al. characterizes the proteomes of the breast muscle in layer chickens at specific time points from 1 to 27 days after hatching (Doherty et al., 2004). In addition, María et al. use the proteome to analyze the tenderness, intramuscular fat, and color lightness of foal meat (López-Pedrouso et al., 2023). Consequently, it can be concluded that proteomics is an effective tool for investigating meat quality traits. However, the influence of age on protein biomarkers linked to meat flavor and nutrition in ducks has not yet been characterized.

In the present study, the breast muscles of Shaoxing White ducks (60- and 900-day-old) were selected to detect the odor and taste, amino acid (AA), free fatty acid (FAA), and nucleotide compositions. Proteomic analysis was also performed to identify candidate protein biomarkers related to meat flavor and nutrition between young and older ducks. This information will provide new insights to explore the biological mechanisms and characterize biomarkers associated with meat flavor in older ducks.

## 2 Materials and methods

### 2.1 Ethics approval

The animals used in this study were raised and euthanized in accordance with the National Standard Guidelines for the Ethical Review of Animal Welfare (GB/T 35892–2018) issued by the General Administration of Quality Supervision, Inspection, and Quarantine of the People's Republic of China and the Standardization Administration of the People's Republic of China. All experimental procedures were approved by the Institute of Animal Husbandry and Veterinary Science of the Zhejiang Academy of Agricultural Sciences (Hangzhou, China). All efforts were made to minimize animal suffering.

### 2.2 Animals and tissue collection

Twelve pure female White Shaoxing ducks (*Anas platyrhynchos*) were obtained from Zhuji Guowei Technology Co., Ltd. (Shaoxing, China), including six 60-day-old ducks (D60) and six 900-day-old ducks (D900). All the ducks were raised under the same conditions, including the water, temperature, and diet. At 60 and 900 days, the ducks were anesthetized with pentobarbital sodium (intraperitoneal injection: 150 mg/kg) and killed by exsanguination. The breast muscle samples were collected, immediately frozen in liquid nitrogen, and stored at −80 °C for proteomic analysis. The remaining breast muscles were cooked for 15 min in boiling water, scooped out, cooled down to room temperature, and stored at 4 °C for nutrient profile analysis.

### 2.3 Label-free quantitative proteomics analysis

The proteomic analysis of duck breast meat was analyzed using the method described by Gu et al. (Gu et al., 2024). Briefly, protein was treated with 100 mM TEAB (Sigma, California, USA), and the mixture was digested overnight. Then the protein was digested, desalted on a C18 SPE column (Nest Group, Southborough, USA), and separated using a NanoElute UHPLC system (Bruker Dal-tonics, Massachusetts, USA) for liquid chromatography-tandem mass spectrometry (LC–MS/MS) analysis. The LC-MS/MS data were used for searches against *Anas platyrhynchos* proteome reference. Fold change (FC) > 1.5 or < 0.67 and a *p*-value <0.05 (*t*-test) were set as the thresholds to determine significant DEPs. Gene Ontology (GO) and Kyoto Encyclopedia of Genes and Genomes (KEGG) annotation were considered functional enrichment analysis.

### 2.4 Discrimination of odor and taste

The odors and tastes of duck breast meat were analyzed using the method described by Song et al. (Song et al., 2013). Briefly, for e-nose analysis, cooked breast sample (5 g) was incubated at 40 °C for 30 min. Then after, headspace sampling was performed, including an injection flow rate and time of 200 mL/min and 3 s, a detection interval of 1 s, and a cleaning time of 120 s, respectively. Principal component analysis (PCA) was performed for 256–260 s using an electronic nose (PEN3, Airsense, Schwerin, Germany). Each odor determination was repeated three times.

For e-tongue analysis, cooked duck meat (10 g) was weighed and homogenized, and the mixture was centrifuged at 9000 rpm for 4 °C, 10 min to collect the supernatant. The supernatant was then filtered using qualitative filter paper. The filtrate was analyzed using e-tongue (TS-SA402B, INSENT, Japan), including acquisition for 120 s and sensor cleaning for 300 s. Following this, six samples per group were evaluated using the e-tongue's proprietary program to determine the following flavors: sourness, bitterness, astringency, aftertaste-bitterness, aftertaste-astringency, umami, richness, saltiness, and sweetness. Each determination was repeated three times.

### 2.5 Analysis of amino acids

An automatic amino acid analyzer (LA 8080, Hitachi, Japan) was used to determine the amino acid composition of cooked breast meat. The procedures were performed according to Weng et al. (Weng et al., 2022).

### 2.6 Analysis of free fatty acids (FFAs)

The crushed meat samples were homogenized in a chloroform–methanol–H_2_O solution (2:1:0.8, *v*/v/v) at 3000 rpm for 10 min. The supernatant was filtered using qualitative filter paper. The filtrate was washed with NaCl (7.3 g/L) and CaCl2 (0.5 *g*/L) solutions and centrifuged at 4000 ×*g* for 15 min. The total lipids were obtained using a rotary evaporator at 44 °C to evaporate the lower phase. Subsequently, the lipids were dissolved in chloroform and transferred onto a pre-activated aminopropyl silica gel cartridge. The gel cartridge was washed with chloroform/2-propanol (2:1, *v*/v), and FFAs were eluted with 2 % acetic acid and dried under nitrogen. The dried products were redissolved in hexane for gas chromatography–mass spectrometry analysis.

### 2.7 Analysis of nucleotides

Cooked meat samples were mixed with 10 mL of 10 % perchloric acid in a glass tube, crushed for 30 min using an ultrasonic processor, and centrifuged at 4000 ×*g* for 15 min to obtain the supernatant. Subsequently, NaOH solution was added to adjust the pH to 6.5. Finally, the solution was filtered through a 0.22 μm membrane filter into an autosampler, and nucleotide composition analysis was then performed.

### 2.8 Statistical analysis

Statistically significant differences between the D60 and D900 groups were determined using a *t*-test. Statistical analyses were performed using GraphPad Prism software (version 8.0; California, USA). The value of *p* < 0.05 and *p* < 0.01 was considered statistically significant and extremely significant, respectively.

## 3 Results

### 3.1 Effect of duck age on odor evaluations of breast meat

**Fig. 1:**
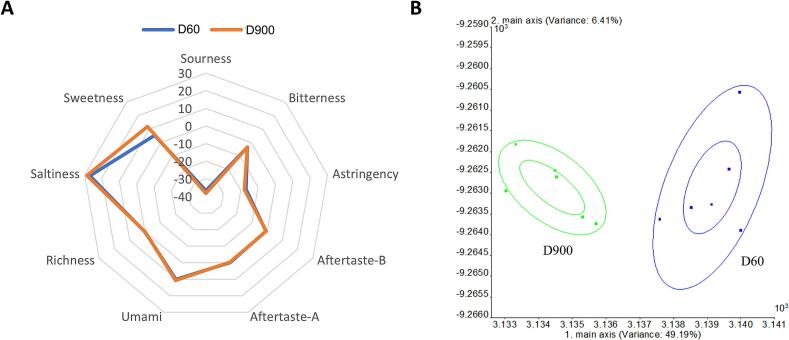
Comparison of the (A) odor profiles and (B) electronic nose principal component analysis (PCA) of cooked duck breasts between different ages. D60, 60-day-old ducks; D900, 900-day-old ducks.

To assess the flavor differences between young and older ducks, an odor sensory evaluation of cooked duck breast was carried out to assess aroma variations. As shown in Fig.1A, the sweetness of the D900 group was intense, however, the odor components were not significantly different. An electronic nose was used to discriminate the aromas of the different groups, and the PCA data are shown in Fig.1B. The first and second principal components (PC1 and PC2) were 49.19 % and 6.41 %, respectively, indicating that the aromas of the two cooked duck meat samples were completely separated.

### 3.2 Effect of age on the amino acid composition of duck breast meat

The free amino acid contents of duck breast meat at 60 and 900 days of age were determined (Table 1). The results showed that the compositions of the total amino acids in the different age groups did not change. As expected, the contents of Asp (umami) and Thr (sweet) in the D900 group were significantly higher than those in the D60 group. Different ages had no effect on the Glu, Arg, Leu, Phe, Ile, Tyr, His, Met, Pro, Ser, Gly, Lys, Val, Ala, and Cys.

**Table 1:** The contents of amino acid in cooked duck breast meat among D60 and D900 groups.

Amino acids	Taste attributes	Contents (g/100 g meat sample)	
		D60	D900
Glu	Umami	2.45 ± 0.14	2.72 ± 0.31
Asp*	Umami	1.72 ± 0.08^b^	1.91 ± 0.13^a^
Arg	Bitter	1.25 ± 0.06	1.29 ± 0.07
Leu*	Bitter	1.73 ± 0.08	1.82 ± 0.09
Phe*	Bitter	0.82 ± 0.02	0.84 ± 0.03
Ile*	Bitter	1.00 ± 0.06	1.06 ± 0.06
Tyr	Bitter	0.70 ± 0.03	0.74 ± 0.03
His	Bitter	0.48 ± 0.03	0.49 ± 0.04
Met*	Bitter	0.48 ± 0.03	0.45 ± 0.06
Pro	Sweet	0.65 ± 0.02	0.65 ± 0.06
Ser	Sweet	0.62 ± 0.04	0.69 ± 0.06
Gly	Sweet	0.90 ± 0.06	0.96 ± 0.07
Lys*	Sweet	1.80 ± 0.09	1.93 ± 0.11
Val*	Sweet	1.10 ± 0.08	1.18 ± 0.08
Thr*	Sweet	0.91 ± 0.04^b^	0.98 ± 0.05^a^
Ala	Sweet	1.43 ± 0.11	1.52 ± 0.14
Cys	–	0.15 ± 0.02	0.15 ± 0.01
TAA		18.17 ± 0.91	19.40 ± 0.86

Note: * represent essential amino acids. ^a,b^Different letters within same row mean significant differences (*p* < 0.05). The same is below.

Glu, glutamic acid; Asp, aspartic acid; Arg, arginine; Pro, proline; Ser, serine; Gly, glycine; Leu, leucine; Lys, lysine; Val, valine; Phe, phenylalanine; Ile, isoleucine; Tyr, tyrosine; His, histidine; Met, methionine; Thr, threonine; Ala, alanine; Cys, cystine; TAA, total amino acids.

### 3.3 Effect of age on nucleotide composition of duck breast meat

The nucleotide compositions of duck breast meat from young and older ducks are shown in Table 2. Significant differences were observed in the nucleotide contents, including those of cytidine-5′-monophosphate (CMP), uridine-5′-monophosphate (UMP), guanosine-5′-monophosphate (GMP), adenosine-5′-monophosphate (AMP) and inosine-5′-monophosphate (IMP) between young and older ducks. The contents of CMP and UMP in duck breast meat collected from the D900 group were significantly lower than those in the D60 group (*p* < 0.05). Meanwhile, GMP and IMP levels in the D900 group were significantly higher than those in the D60 group (*p* < 0.05). Interestingly, AMP content in the D900 group was undetectable.

**Table 2:** The contents of nucleotide in cooked duck breast meat among D60 and D900 groups.

Nucleotide	Contents (mg/kg)	
	D60	D900
CMP	13.29 ± 1.95^a^	2.92 ± 0.17^b^
UMP	27.68 ± 3.43^a^	10.66 ± 5.45^b^
GMP	40.64 ± 12.66^b^	312.02 ± 118.35^a^
AMP	158.99 ± 39.39	–
IMP	132.95 ± 8.16^b^	153.07 ± 17.44^a^

CMP: cytidine-5′-monophosphate; UMP: uridine-5′-monophosphate; GMP: guanosine-5′-monophosphate; AMP: adenosine-5′-monophosphate; IMP: inosine-5′- monophosphate. “–” represented undetected. Data are shown as mean ± standard deviation (*n* = 5), and different letters showed significant differences (*p* < 0.05).

### 3.4 Effect of age on fatty acid compositions of duck breast meat

Variations in the fatty acid compositions of duck breast meat were also determined in young and older ducks. Sixteen FFAs were detected in ducks (Table 3), including six saturated fatty acids (SFAs), four monounsaturated fatty acids (MUFAs), and six polyunsaturated fatty acids (PUFAs). Considering meat products from different aged ducks, the breast meat at D900 group had a significantly higher MUFA (C18:1 t) content and a lower SFA (C14:0 and C18:0) content compared to those at D60 group. There were no significant differences in PUFA levels between the D60 and D900 groups, whereas breast meat from D900 group had a significantly higher C18:2 content and lower C18:3 and C22:6 contents compared to those in the D60 group.

**Table 3:** The contents of free fatty acid in cooked duck breast meat among D60 and D900 groups.

FFA (%)	D60	D900
C14:0	0.72 ± 0.20^a^	0.33 ± 0.17^b^
C16:0	24.93 ± 0.92	19.00 ± 8.20
C17:0	0.00 ± 0.00	0.29 ± 0.65
C18:0	19.56 ± 0.68^a^	11.83 ± 5.65^b^
C20:0	0.24 ± 0.21	0.31 ± 0.25
C22:0	0.31 ± 0.17	0.17 ± 0.10
C16:1	1.03 ± 0.25	1.26 ± 0.63
C18:1	0.00 ± 0.00	0.21 ± 0.52
C18:1 t	18.03 ± 0.51^b^	30.49 ± 5.61^a^
C22:1	0.41 ± 0.35	0.41 ± 0.43
C18:2	10.69 ± 0.62^b^	13.84 ± 2.52^a^
C18:3	1.25 ± 0.22^a^	0.68 ± 0.17^b^
C20:3	0.00 ± 0.00	0.06 ± 0.16
C20:4	20.85 ± 1.85	19.60 ± 4.45
C20:5	0.00 ± 0.00	0.07 ± 0.18
C22:6	2.00 ± 0.12^a^	1.30 ± 0.20^b^
SFA	45.75 ± 1.51^a^	31.93 ± 13.20^b^
UFA	54.25 ± 1.51^b^	68.07 ± 13.20^a^
MUFA	19.47 ± 0.55^b^	32.38 ± 5.94^a^
PUFA	34.78 ± 1.48	35.69 ± 7.46

FFA, free fatty acid; SFA, saturated fatty acid; UFA, unsaturated fatty acid; MUFA, monounsaturated fatty acid; PUFA, polyunsaturated fatty acid.

### 3.5 Comparative analysis of breast meat proteomes from young and older ducks

**Fig. 2:**
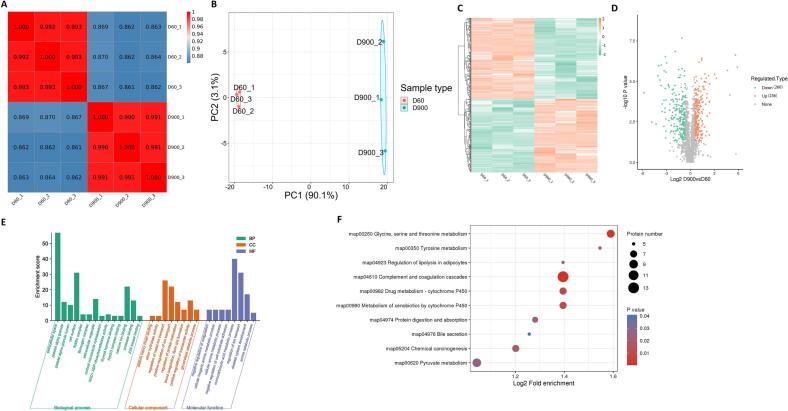
Proteomic profiles of breast meat from ducks at D60 and D900 ducks. (A) Correlation analysis and (B) principal component analysis (PCA) of the samples from D60 and D900 groups. (C) Heatmap analysis and (D) volcano plot of differentially expressed proteins (DEPs). Orange and green points indicate the proteins for which expression was significantly upregulated and downregulated, respectively; gray points indicate the proteins with no significant differences. (E) GO enrichment. (F) KEGG pathway analysis. (For interpretation of the references to color in this figure legend, the reader is referred to the web version of this article.)

To further investigate the mechanisms underlying flavor differences between young and older ducks, we performed proteomic sequencing. As shown in Fig.2A and B, correlation analysis and PCA data were clustered within their own groups, indicating that the discrimination was more obvious. In total, 496 DEPs between the D60 and D900 groups (260 downregulated and 236 upregulated proteins) were tested (Fig.2C and D). To gain insight into the biological functions of the groups, we performed GO and KEGG analyses based on the DEPs. GO analysis revealed the enrichment of many terms, of which the top enriched terms were mostly related to the extracellular space, monocarboxylic acid metabolism, and the regulation of ion transport (Fig.2E). KEGG analysis of the DEPs reflected the enrichment of glycine, serine, and threonine metabolism, tyrosine metabolism and pyruvate metabolism (Fig.2F and Table 4), which are known to be associated with meat flavor.

**Table 4:** The key protein of regulated flavor and nutrition and their involved signaling pathways in D900 compared with D60 group.

Accession	Protein	Gene name	Group radio (D900/D60)	Signaling pathway
R0KDI6	Dimethylglycine dehydrogenase	DMGDH	0.035	glycine, serine and threonine metabolism
R0M714	Betaine–homocysteine *S*-methyltransferase 1	BHMT	0.051	glycine, serine and threonine metabolism
R0JWP0	Choline dehydrogenase	CHDH	0.088	glycine, serine and threonine metabolism
R0LQ88	O-phosphoserine phosphohydrolase	PSPH	0.647	glycine, serine and threonine metabolism
R0LFM5	Aldehyde dehydrogenase (NAD(+))	ALDH7A1	0.624	glycine, serine and threonine metabolism/ pyruvate metabolism
R0KYW6	Phosphoglycerate mutase 1	BPGM	1.575	glycine, serine and threonine metabolism
R0L177	Glyoxylate reductase/hydroxypyruvate reductase	GRHPR	2.321	glycine, serine and threonine metabolism/ pyruvate metabolism
R0M1F3	Amine oxidase [flavin-containing] A	MAOA	0.636	glycine, serine and threonine metabolism/ tyrosine metabolism
R0KUJ7	Aspartate aminotransferase	GOT2	1.503	tyrosine metabolism
R0L8P5	S-(hydroxymethyl) glutathione dehydrogenase	ADH5	0.664	tyrosine metabolism
R0JKM2	Catechol *O*-methyltransferase	COMT	1.571	tyrosine metabolism
R0JLZ8	Macrophage migration inhibitory factor	MIF	1.585	tyrosine metabolism
R0JMD9	Hydroxyacylglutathione hydrolase	HAGH	1.605	pyruvate metabolism
R0LEM6	Malic enzyme 2	ME2	0.653	pyruvate metabolism
R0LYG1	Acylphosphatase 1	ACYP1	2.77	pyruvate metabolism
R0KQL3	Malic enzyme 3	ME3	0.289	pyruvate metabolism
R0L871	Acylphosphatase-2	ACYP2	1.682	pyruvate metabolism
R0LAA8	Lactoylglutathione lyase	GLO1	1.601	pyruvate metabolism
R0L734	Pyruvate dehydrogenase E1 component subunit beta	PDHB	1.503	pyruvate metabolism

### 3.6 Proteomic analysis related to meat flavor (amino acids, nucleotides, and FFAs) of young and older ducks

The prediction of meat flavor and nutrition using protein biomarkers was currently of great importance. To estimate the relationship between meat flavor and protein abundance in different aged ducks, pearson correlation coefficients were determined. The correlation coefficients for meat flavor, including amino acids, nucleotides, and FFAs between D60 and D900 ducks were shown in Table 5. Our results showed a significant association among amino acids (Asp and Thr), nucleotides (CMP, UMP, GMP, AMP, and IMP), FFAs (C18:0, C18:1 t, C18:2, C18:3, C22:6, SFA, UFA, and MUFA) and 13 proteins in 60-day-old ducks. Among them, significant correlations between Asp and PDHB (*r* = −1.000), DMGDH (*r* = 1.000) were observed. Consistent with these observations, significant correlations between nucleotides (CMP, UMP, GMP, AMP, and IMP) and ADH5 (*r* = 1.000), GLO1 (r = 1.000), MAOA (*r* = 0.998), ME2 (r = ˗1.000) were observed in 60-day-old ducks. For FFAs, significant correlations were observed with PDHB (*r* = −0.999), CHDH (*r* = 0.999), ME2 (r = 0.998), ME3 (*r* = −0.998), BPGM (r = 1.000), ALDH7A1 (r = 0.998), MIF (r = 1.000), HAGH (r = −0.999) in 60-day-old ducks.

**Table 5:** Correlation coefficients (r) of differential abundant proteins with meat flavor in D60 and D900 ducks.

Items		Asp	Thr	CMP	UMP	GMP	AMP	IMP	C18:0	C18:1 t	C18:2	C18:3	C22:6	SFA	UFA	MUFA
D60	DMGDH	0.768	1.000*	0.539	−0.640	−0.462	−0.329	0.150	−0.779	0.538	−0.239	−0.654	0.879	−0.948	0.949	0.221
	BHMT	0.997	0.736	−0.205	−0.995	−0.951	−0.896	−0.585	−0.995	0.974	0.509	0.062	0.294	−0.900	0.899	0.841
	CHDH	0.963	0.593	−0.387	−0.996	−0.992	−0.964	−0.728	−0.958	0.999*	0.663	0.251	0.107	−0.800	0.799	0.928
	PSPH	0.481	0.919	0.809	−0.315	−0.106	0.039	0.501	−0.496	0.192	−0.577	−0.885	0.992	−0.766	0.767	−0.150
	ALDH7A1	−0.951	−0.940	−0.188	0.879	0.758	0.655	0.227	0.956	−0.812	−0.138	0.327	−0.640	0.998*	−0.998*	−0.567
	BPGM	0.357	0.857	0.882	−0.183	0.030	0.175	0.614	−0.373	0.057	−0.683	−0.940	1.000**	−0.672	0.673	−0.283
	GRHPR	0.664	0.062	−0.825	−0.788	−0.901	−0.954	−0.983	−0.651	0.859	0.963	0.736	−0.449	−0.347	0.346	0.982
	MAOA	−0.993	−0.710	0.242	0.998*	0.962	0.912	0.615	0.991	−0.982	−0.541	−0.100	−0.258	0.883	−0.882	0.861
	GOT2	−0.981	−0.892	−0.069	0.930	0.831	0.741	0.342	0.984	−0.876	−0.256	0.211	−0.543	0.984	−0.984	−0.662
	ADH5	−0.143	0.496	1.000*	0.320	0.514	0.633	0.921	0.126	−0.437	−0.953	−0.987	0.866	−0.225	0.227	−0.715
	COMT	−0.315	0.337	0.981	0.481	0.656	0.759	0.975	0.299	−0.588	−0.991	−0.943	0.756	−0.051	0.052	−0.827
	MIF	0.785	0.236	−0.714	−0.884	−0.963	−0.992	−0.937	−0.774	0.936	0.901	0.606	−0.286	−0.506	0.505	1.000*
	HAGH	0.946	0.945	0.203	−0.872	−0.748	−0.644	−0.212	−0.951	0.803	0.123	−0.341	0.651	−0.999*	−0.999*	0.555
	ME2	0.933	0.514	−0.473	0.983	−1.000*	−0.985	−0.790	−0.927	0.998*	0.731	0.342	0.012	−0.740	0.739	0.959
	ACYP1	0.983	0.888	0.060	−0.933	−0.836	−0.748	−0.351	−0.986	0.881	0.265	−0.202	0.535	−0.983	0.982	0.669
	ME3	−0.044	0.580	0.997	0.224	0.426	0.552	0.878	0.027	−0.345	−0.918	−0.998*	0.912	−0.321	0.323	−0.642
	ACYP2	0.789	0.244	−0.708	−0.887	−0.965	−0.993	−0.934	−0.779	0.938	0.898	0.599	−0.278	−0.513	0.512	1.000*
	GLO1	−0.116	0.520	1.000**	0.294	0.490	0.611	0.910	0.099	−0.412	−0.944	−0.991	0.880	−0.252	0.253	−0.696
	PDHB	−1.000*	−0.777	0.143	0.987	0.929	0.866	0.533	−0.999*	−0.958	−0.454	0.000	−0.354	0.925	−0.925	−0.805
D900	DMGDH	0.387	0.006	0.949	−0.604	−0.455	–	−1.000*	−0.999*	−0.999*	1.000*	0.194	0.912	−1.000*	1.000*	0.998*
	BHMT	−0.522	−0.158	−0.890	0.718	0.585	–	0.986	0.993	−0.994	−0.989	−0.341	−0.964	0.992	−0.992	−0.995
	CHDH	−0.395	−0.015	−0.946	0.611	0.463	–	1.000*	1.000*	−0.999*	−1.000**	−0.203	−0.916	1.000*	−1.000*	−0.999*
	PSPH	0.648	0.309	0.808	−0.817	−0.704	–	−0.948	−0.963	0.965	0.954	0.482	0.993	-0.961	0.961	0.968
	ALDH7A1	−1.000*	0.912	−0.106	0.976	0.999*	–	0.401	0.447	−0.455	−0.418	−0.973	−0.752	0.440	−0.440	−0.464
	BPGM	−0.901	−0.668	−0.501	0.891	0.931	–	0.738	0.771	−0.777	−0.751	−0.796	−0.955	0.767	−0.767	−0.784
	GRHPR	−0.695	−0.369	−0.769	0.852	0.748	–	0.926	0.944	−0.947	−0.933	−0.537	−0.999*	0.941	−0.941	−0.950
	MAOA	0.998*	0.945	0.018	−0.953	−0.991	–	−0.319	−0.366	0.374	0.336	0.990	0.690	−0.359	0.359	0.384
	GOT2	0.730	0.936	−0.626	−0.537	−0.677	–	0.362	0.315	−0.306	−0.345	0.852	0.067	0.321	−0.322	−0.296
	ADH5	−0.503	−0.795	0.824	0.272	0.437	–	−0.614	−0.574	0.567	0.600	−0.666	0.222	−0.580	0.580	0.558
	COMT	0.896	0.998*	−0.375	−0.757	−0.860	–	0.078	0.029	−0.019	−0.060	0.967	0.351	0.036	−0.036	−0.008
	MIF	0.994	0.876	0.186	−0.990	−0.999*	–	−0.474	−0.517	0.525	0.490	0.951	0.802	−0.511	0.511	0.534
	HAGH	0.999*	0.907	0.118	−0.978	−0.999*	–	−0.412	−0.457	0.465	0.429	0.970	0.760	−0.451	0.451	0.475
	ME2	0.538	0.819	−0.800	−0.312	−0.473	–	0.581	0.540	−0.532	−0.566	0.696	−0.182	0.546	−0.546	−0.523
	ACYP1	0.577	0.223	0.858	−0.762	−0.637	–	−0.973	−0.983	0.985	0.977	0.402	0.979	−0.982	0.982	0.987
	ME3	0.265	−0.122	0.981	−0.497	−0.337	–	0.994	−0.987	0.985	0.991	0.067	0.852	−0.988	0.988	0.983
	ACYP2	−0.756	−0.449	−0.710	0.895	0.803	–	0.889	0.911	−0.915	−0.898	−0.609	−0.999*	0.908	−0.908	−0.919
	GLO1	−0.758	−0.950	0.592	0.572	0.707	–	−0.322	−0.274	0.265	0.304	−0.874	−0.110	−0.281	0.281	0.255
	PDHB	0.108	0.479	−0.983	0.143	−0.033	–	0.882	0.587	−0.853	−0.873	0.305	−0.599	0.861	−0.861	−0.847

Note: Levels of significance: *p < 0.05; **p < 0.01.”—” represented that AMP was not been detected in D900 group.

Although a strong relationship between meat flavor and protein abundance was observed in 60-day-old ducks, a similar relationship was also observed in 900-day-old ducks. Significant correlations were observed between the amino acid parameters and ALDH7A1 (*r* = −1.000), MAOA (r = 0.998), HAGH (r = 0.999), COMT (r = 0.998). Some proteins were also significantly associated with nucleotides, such as ALDH7A1 (r = 0.999), MIF (r = −0.999), DMGDH (r = −1.000), and CHDH (r = 1.000). Furthermore, many proteins, including DMGDH (r = −0.999), CHDH (r = 1.000), GRHPR (r = −0.999), and ACYP2 (r = −0.999), were highly associated with FFAs. Interestingly, six proteins, BPGM, ADH5, ME2, ME3, GLO1, and PDHB, were specifically correlated with meat flavor in 60-day-old-duck meat, whereas only two proteins, GRHPR and COMT, showed a specific correlation with meat flavor in 900-day-old-duck meat.

## 4 Discussion

In recent years, China has become the most productive duck meat producer worldwide, and duck meat has become popular among consumers (Zeng et al., 2016). Age is closely related to meat nutrition and flavor in poultry production. In the present study, we evaluated the differences in meat nutrition and flavor between 60- and 900-day-old ducks and determined how proteins influence meat nutrition in ducks of different ages based on proteomics.

The e-nose, which mimics human senses and requires no reagents, is widely recognized for its precision and accuracy in distinguishing flavor differences in samples (Zaukuu et al., 2020). In the present study, we conducted an e-nose analysis of different aged duck meat. The PCA showed that the e-nose effectively distinguished meat from 60- and 900-day-old ducks, which indicated that the meat of different ages has significant differences in volatile components (Cui et al., 2015).

Free amino acids are the end products of protein degradation (Je et al., 2005) and contribute to the favorable taste of meat products (Lorenzo & Franco, 2012). In addition, the amount of amino acids in meat directly affects its nutritional value (Lund et al., 2011; Zhang et al., 2013). We observed that the Asp (umami) and Thr (sweet) levels in the D900 group were significantly higher than those in the D60 group (*p* < 0.05). These amino acids not only increase the nutritional value but also cause changes in the flavor-associated compounds of cooked meat (Zhang et al., 2010). This indicates that 900-day-old duck meat can release Asp and Thr to promote the umami taste and improve the sweet taste to a greater extent than 60-day-old duck meat. Furthermore, IMP and GMP are key compounds that contribute to flavor (Katemala et al., 2021), participating in energy metabolism, and ensuring an energy supply for cells. IMP is generated by adenosine triphosphate consumption (Zhang et al., 2018) and can be converted to GMP (Peifer et al., 2012). Additionally, evidences have shown that adenylate content in meat may continue to degrade beyond AMP to other adenylate homologs including IMP during biological tissue age (Aliani et al., 2013; Smith et al., 2024). In our study, 900-day-old ducks had higher IMP and GMP contents than 60-day-old ducks, while the AMP contents in 900-day-old ducks were undetectable. In addition, Lioe et al. (Lioe et al., 2006) and Wood et al. (Wood et al., 2004) report that IMP and GMP contribute to meat flavor perceptions and together comprise the umami taste, which was similar to our finding that the Asp content was higher in 900-day-old ducks than in 60-day-old ducks.

FFAs are released through the hydrolysis of triglycerides and phospholipids (Huang et al., 2014; Ying et al., 2016), and the presence of UFAs in meat is thought to be beneficial for humans (Wood et al., 2004). In this study, the SFA (C14:0 and C18:0) content was significantly decreased, while the MUFA (C18:1 t) and PUFA (C18:2) contents were significantly increased with advanced age, indicating that higher MUFA and PUFA contents could be beneficial for duck meat and contribute to nutrition and flavor. Our results were similar to those of a previous study on geese (Weng et al., 2022), which reported that 300-day-old geese contained higher C18:1n9c and C18:2n6c contents than 70-day-old geese. Additionally, the lower SFA and higher UFA and MUFA contents in 900-day-old ducks could be associated with improved meat flavor (Ponnampalam et al., 2012). Overall, older duck meat has advantages in terms of amino acid, nucleotide, and FFA compositions. Umami amino acids, sweet amino acids, IMP, GMP, UFA, and MUFAs were found to be deposited with increasing age.

The proteomic approach is a powerful technique for studying protein changes and has been widely used to identify potential protein expression in food science (López-Pedrouso et al., 2023; Schilling et al., 2017; J. Zhang et al., 2021). Mekchay et al. characterized the muscle proteome to determine the relationship between native Thai and commercial broiler chickens (Mekchay et al., 2010). Protein expression was also investigated in the breast muscles of Ross 708 commercial broilers and Leghorn chicks to distinguish between the chicken genotypes (Zapata et al., 2012). In this study, an LC–MS/MS-based proteomic analysis of breast muscle was performed that 496 DEPs were screened between young and older ducks. These DEPs were mainly associated with glycine, serine, threonine, tyrosine, and pyruvate metabolism. The results of this study strengthen our knowledge of protein expression and provide a marker for distinguishing young duck meat from older duck meat.

Among the 19 annotated proteins obtained based on glycine, serine, threonine, tyro-sine, and pyruvate metabolism, six proteins were of interest, specifically because BPGM, ADH5, ME2, ME3, GLO1, PDHB, GRHPR, and COMT are associated with amino acids, nucleotides, and FFAs in 60-day-old and 900-day-old duck meat. PDHB catalyzes the conversion of pyruvate to acetyl-CoA and is involved in fatty acid oxidation, acting as a central node linking glucose metabolism, lipid metabolism, and the TCA (Patel et al., 2014). BPGM participates in the production of 2,3-bisphosphoglycerate during glycolysis to modulate the downstream processes of glycolysis and serine biosynthetic flux (Oslund et al., 2017; Wei et al., 2015). ADH5 is a longevity-associated gene that can remove damaged mitochondria and limit cellular deterioration to promote the anti-aging process (Rizza & Filomeni, 2018). In addition, changes in GLO1 not only affect dicarbonyl and oxidative stress but also lipid and glucose metabolism (Šilhavý et al., 2020). In addition, COMT expression is significantly reduced in obese mice fed a high-fat diet, and low activity of the COMT allele is associated with abdominal obesity (Bozek et al., 2017; Kanasaki et al., 2017). We inferred that the protein expression of BPGM, ADH5, ME2, ME3, GLO1, PDHB, GRHPR, and COMT could have a role in distinguishing between young and older ducks and in regulating meat nutrition and flavor deposition.

## 5 Conclusions

In conclusion, the present study suggests that 900-day-old duck has superior nutrition and flavor compared to 60-day-old ducks in terms of amino acids, nucleotides, and FFAs. The proteomic approach used in this study revealed significant age-related changes and differences at the muscle level. Furthermore, proteomic shifts related to amino acids, nucleotides, and FFAs revealed several candidate protein biomarkers, such as BPGM, ADH5, ME2, ME3, GLO1, PDHB, GRHPR, and COMT, for the authentication of older ducks, which require further investigation and validation.

## CRediT authorship contribution statement

**Tiantian Gu:** Writing – review & editing, Writing – original draft, Investigation, Data curation. **Mingcai Duan:** Validation, Methodology. **Li Chen:** Formal analysis. **Yong Tian:** Methodology, Data curation. **Wenwu Xu:** Software. **Tao Zeng:** Project administration. **Lizhi Lu:** Writing – review & editing, Project administration.

## Declaration of competing interest

The authors declare that they have no known competing financial interests or personal relationships that could have appeared to influence the work reported in this paper.

## Data Availability

Data will be made available on request.

## References

[bb0005] Aliani M., Farmer L.J., Kennedy J.T., Moss B.W., Gordon A. (2013). Post-slaughter changes in ATP metabolites, reducing and phosphorylated sugars in chicken meat. Meat Science.

[bb0010] Bai H., Bao Q., Zhang Y., Song Q., Liu B., Zhong L., Chen G. (2020). Research note: Effects of the rearing method and stocking density on carcass traits and proximate composition of meat in small-sized meat ducks. Poultry Science.

[bb0015] Bozek T., Blazekovic A., Perkovic M.N., Jercic K.G., Sustar A., Smircic-Duvnjak L., Borovecki F. (2017). The influence of dopamine-beta-hydroxylase and catechol O-methyltransferase gene polymorphism on the efficacy of insulin detemir therapy in patients with type 2 diabetes mellitus. Diabetology and Metabolic Syndrome.

[bb0020] Cui S., Wang J., Yang L., Wu J., Wang X. (2015). Qualitative and quantitative analysis on aroma characteristics of ginseng at different ages using E-nose and GC-MS combined with chemometrics. Journal of Pharmaceutical and Biomedical Analysis.

[bb0025] Doherty M.K., McLean L., Hayter J.R., Pratt J.M., Robertson D.H., El-Shafei A., Beynon R.J. (2004). The proteome of chicken skeletal muscle: Changes in soluble protein expression during growth in a layer strain. Proteomics.

[bb0030] Gu T., Duan M., Chen L., Tian Y., Xu W., Zeng T., Lu L. (2024). Proteomic-metabolomic combination analysis reveals novel biomarkers of meat quality that differ between young and older ducks. Poultry Science.

[bb0035] Huang Y., Li H., Huang T., Li F., Sun J. (2014). Lipolysis and lipid oxidation during processing of Chinese traditional smoke-cured bacon. Food Chemistry.

[bb0040] Je J.Y., Park P.J., Jung W.K., Kim S.K. (2005). Amino acid changes in fermented oyster (Crassostrea gigas) sauce with different fermentation periods. Food Chemistry.

[bb0045] Kanasaki M., Srivastava S.P., Yang F., Xu L., Kudoh S., Kitada M., Koya D. (2017). Deficiency in catechol-o-methyltransferase is linked to a disruption of glucose homeostasis in mice. Scientific Reports.

[bb0050] Katemala S., Molee A., Thumanu K., Yongsawatdigul J. (2021). Meat quality and Raman spectroscopic characterization of Korat hybrid chicken obtained from various rearing periods. Poultry Science.

[bb0055] Lioe H.N., Apriyantono A., Takara K., Wada K., Yasuda M. (2006). Umami taste enhancement of MSG/NaCl mixtures by subthreshold L-a-aromatic amino acids. Journal of Food Science.

[bb0060] Liu C., Pan D., Ye Y., Cao J. (2013). ^1^H NMR and multivariate data analysis of the relationship between the age and quality of duck meat. Food Chemistry.

[bb0065] López-Pedrouso M., Franco D., Serrano M.P., Maggiolino A., Landete-Castillejos T., De Palo P., Lorenzo J.M. (2019). A proteomic-based approach for the search of biomarkers in Iberian wild deer (Cervus elaphus) as indicators of meat quality. Journal of Proteomics.

[bb0070] López-Pedrouso M., Lorenzo J.M., Cittadini A., Sarries M.V., Gagaoua M., Franco D. (2023). A proteomic approach to identify biomarkers of foal meat quality: A focus on tenderness, color and intramuscular fat traits. Food Chemistry.

[bb0075] Lorenzo J.M., Franco D. (2012). Fat effect on physico-chemical, microbial and textural changes through the manufactured of dry-cured foal sausage lipolysis, proteolysis and sensory properties. Meat Science.

[bb0080] Lund M.N., Heinonen M., Baron C.P., Estévez M. (2011). Protein oxidation in muscle foods: A review. Molecular Nutrition & Food Research.

[bb0085] Mekchay S., Teltathum T., Nakasathien S., Pongpaichan P. (2010). Proteomic analysis of tenderness trait in Thai native and commercial broiler chicken muscles. The Journal of Poultry Science.

[bb0090] Oslund R.C., Su X., Haugbro M., Kee J.M., Esposito M., David Y., Rabinowitz J.D. (2017). Bisphosphoglycerate mutase controls serine pathway flux via 3-phosphoglycerate. Nature Chemical Biology.

[bb0095] Patel M.S., Nemeria N.S., Furey W., Jordan F. (2014). The pyruvate dehydrogenase complexes: Structure-based function and regulation. The Journal of Biological Chemistry.

[bb0100] Peifer S., Barduhn T., Zimmet S., Volmer D.A., Heinzle E., Schneider K. (2012). Metabolic engineering of the purine biosynthetic pathway in Corynebacterium glutamicum results in increased intracellular pool sizes of IMP and hypoxanthine. Microbial Cell Factories.

[bb0105] Ponnampalam E.N., Butler K.L., McDonagh M.B., Jacobs J.L., Hopkins D.L. (2012). Relationship between muscle antioxidant status, forms of iron, polyunsaturated fatty acids and functionality (retail colour) of meat in lambs. Meat Science.

[bb0110] Rizza S., Filomeni G. (2018). Denitrosylate and live longer: How ADH5/GSNOR links mitophagy to aging. Autophagy.

[bb0115] Schilling M.W., Suman S.P., Zhang X., Nair M.N., Desai M.A., Cai K., Allen P.J. (2017). Proteomic approach to characterize biochemistry of meat quality defects. Meat Science.

[bb0120] Šilhavý J., Malínská H., Hüttl M., Marková I., Oliyarnyk O., Mlejnek P., Pravenec M. (2020). Downregulation of the Glo1 gene is associated with reduced adiposity and ectopic fat accumulation in spontaneously hypertensive rats. Antioxidants (Basel).

[bb0125] Smith N.W., Sindelar J.J., Rankin S.A. (2024). AMP, ADP, and ATP concentrations differentially affected by meat processing, manufacturing, and nonmeat ingredients. Journal of Food Protection.

[bb0130] Song S., Yuan L., Zhang X., Hayat K., Chen H., Liu F., Xiao Z., Niu Y. (2013). Rapid measuring and modelling flavour quality changes of oxidised chicken fat by electronic nose profiles through the partial least squares regression analysis. Food Chemistry.

[bb0135] Wei S.N., Zhao W.J., Zeng X.J., Kang Y.M., Du J., Li H.H. (2015). Microarray and co-expression network analysis of genes associated with acute doxorubicin cardiomyopathy in mice. Cardiovascular Toxicology.

[bb0140] Weng K., Huo W., Song L., Cao Z., Zhang Y., Zhang Y., Chen G., Xu Q. (2022). Effect of marketable age on nutritive profile of goose meat based on widely targeted metabolomics. LWT.

[bb0145] Wood J.D., Richardson R.I., Nute G.R., Fisher A.V., Campo M.M., Kasapidou E., Enser M. (2004). Effects of fatty acids on meat quality: A review. Meat Science.

[bb0150] Xiao Z., Ge C., Zhou G., Zhang W., Liao G. (2019). (1)H NMR-based metabolic characterization of Chinese Wuding chicken meat. Food Chemistry.

[bb0155] Ying W., Ya-Ting J., Jin-Xuan C., Yin-Ji C., Yang-Ying S., Xiao-Qun Z., Dao-Dong P., Chang-Rong O., Ning G. (2016). Study on lipolysis-oxidation and volatile flavour compounds of dry-cured goose with different curing salt content during production. Food Chemistry.

[bb0160] Zapata I., Reddish J.M., Miller M.A., Lilburn M.S., Wick M. (2012). Comparative proteomic characterization of the sarcoplasmic proteins in the pectoralis major and supracoracoideus breast muscles in 2 chicken genotypes. Poult Science.

[bb0165] Zaukuu J.L.Z., Bazar G., Gillay Z., Kovacs Z. (2020). Emerging trends of advanced sensor based instruments for meat, poultry and fish quality- a review. Critical Reviews in Food Science and Nutrition.

[bb0170] Zeng T., Chen L., Du X., Lai S.J., Huang S.P., Liu Y.L., Lu L.Z. (2016). Association analysis between feed efficiency studies and expression of hypothalamic neuropeptide genes in laying ducks. Animal Genetics.

[bb0175] Zhang J., Cao J., Geng A., Wang H., Chu Q., Yang L., Liu H. (2021). Comprehensive proteomic characterization of the pectoralis major at three chronological ages in Beijing-you chicken. Frontiers in Physiology.

[bb0180] Zhang T., Lu H., Wang L., Yin M., Yang L. (2018). Specific expression pattern of IMP metabolism related-genes in chicken muscle between cage and free range conditions. PLoS One.

[bb0185] Zhang W., Xiao S., Ahn D.U. (2013). Protein oxidation: Basic principles and implications for meat quality. Critical Reviews in Food Science and Nutrition.

[bb0190] Zhang W., Xiao S., Samaraweera H., Lee E.J., Ahn D.U. (2010). Improving functional value of meat products. Meat Science.

